# Delayed Diagnosis of Thyroid Storm Presenting With Predominant Gastrointestinal Symptoms

**DOI:** 10.7759/cureus.106121

**Published:** 2026-03-30

**Authors:** Tetsuya Kawahara, Mikio Toda, Maiko Kanagawa, Nagahiro Toyama

**Affiliations:** 1 Endocrinology and Diabetes, Shinkomonji Hospital, Kitakyushu, JPN; 2 Emergency Medicine, Shinkomonji Hospital, Kitakyushu, JPN

**Keywords:** diagnostic delay, gastrointestinal symptoms, hyperthyroidism, hyroid storm, superior mesenteric artery syndrome

## Abstract

Thyroid storm is a rare but life-threatening endocrine emergency. Although mortality predictors have been described, diagnostic delay remains under-recognized, particularly in atypical presentations. A previously healthy man in his 20s presented with persistent nausea, vomiting, diarrhea, and 5-kg weight loss over one month. Abdominal computed tomography suggested superior mesenteric artery (SMA) syndrome. Despite supportive treatment, symptoms fluctuated and subsequently worsened with hemodynamic instability. Thyroid function tests performed during readmission revealed markedly elevated free thyroid hormone levels with suppressed TSH. He fulfilled diagnostic criteria for thyroid storm. Despite intensive treatment including antithyroid drugs, beta-blockade, corticosteroids, mechanical ventilation, and extracorporeal membrane oxygenation, he died from progressive multiorgan failure. Thyroid storm may present predominantly with gastrointestinal symptoms and minimal classical features, leading to diagnostic anchoring on radiological findings. Early thyroid function testing should be considered in patients with persistent unexplained gastrointestinal symptoms accompanied by systemic deterioration.

## Introduction

Thyroid storm is a severe and life-threatening condition [1-3]; however, it is potentially reversible if diagnosed early and treated promptly in accordance with established guidelines [4,5]. In contrast, when patients present without classic features such as hyperthermia or neuropsychiatric symptoms, diagnosis may be delayed, even if other manifestations of hyperthyroidism, such as weight loss, diarrhea, vomiting, or liver dysfunction, are present. Without timely initiation of appropriate therapy, the disease can progress to multiorgan failure and result in death [6,7]. We report a fatal case of thyroid storm initially diagnosed as superior mesenteric artery syndrome, underscoring the impact of diagnostic anchoring.

## Case presentation

**Figure 1:**
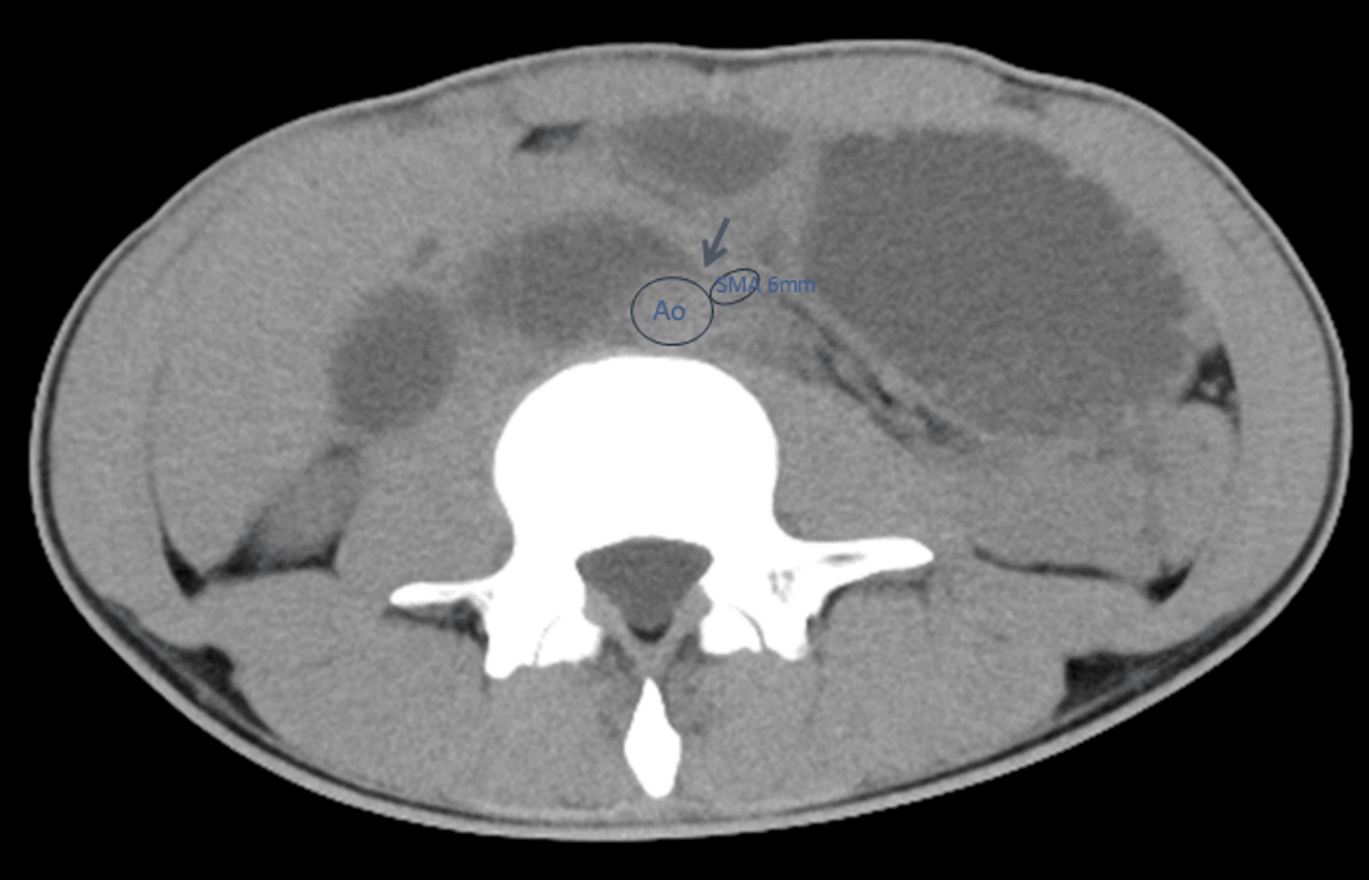
Abdominal computed tomography A bird-beak sign (arrow) was observed at the duodenum, compressed between the aorta and the SMA, accompanied by proximal gastric dilatation. The aortomesenteric distance was 6 mm (≤8 mm), which is the established diagnostic criterion for SMA syndrome. Contrast-enhanced CT was not performed; therefore, the aortomesenteric angle (≤25°), which is also the diagnostic criterion for SMA syndrome, could not be evaluated. Ao, aorta; SMA, superior mesenteric artery.

A previously healthy man in his 20s presented with a one-month history of nausea, vomiting, diarrhea, and a 5-kg weight loss following an episode of influenza, without fever. Abdominal computed tomography (CT) demonstrated narrowing of the duodenum between the aorta and the superior mesenteric artery with proximal gastric dilatation (Figure 1), consistent with suspected superior mesenteric artery (SMA) syndrome [8]. Laboratory findings were unremarkable (Table 1). He was admitted to the hospital, and both upper and lower gastrointestinal endoscopy revealed no abnormalities. Nasogastric tube feeding was initiated, leading to a gradual improvement of his symptoms, and he was discharged after 10 days of hospitalization. However, symptoms recurred, with the development of low-grade fever, and he was readmitted four days after discharge. Repeat CT showed that the previously observed findings had resolved. He was managed with nasogastric tube feeding; however, on this occasion, his symptoms did not improve. As his body temperature and heart rate gradually increased, thyroid function tests were obtained, revealing markedly elevated free T3 (triiodothyronine) (28.3 pg/mL) and free T4 (thyroxine) (10.0 ng/dL) levels with suppressed thyroid-stimulating hormone (TSH) (0.001 μIU/mL). He had no family history of thyroid disease. Based on these findings, a diagnosis of thyrotoxicosis was made, and he was subsequently transferred to our hospital.

**Table 1:** Trends in laboratory data T-Bil, total-bilirubin; AST, aspartate aminotransferase; ALT, alanine aminotransferase; LDH, lactate dehydrogenase; γ-GTP, γ-glutamyl transpeptidase; CPK, creatine phosphokinase; BUN, blood urea nitrogen; TSH, thyroid-stimulating hormone; TRAb, TSH receptor antibody; TsAb, thyroid-stimulating antibody; TgAb, thyroglobulin antibody; TPOAb, thyroid peroxidase antibody; BNP, brain natriuretic peptide; PT-INR, prothrombin time-international normalized ratio.

Test	Day 30 of illness	Day 44 of illness	Day 51 of illness	Day 54 of illness	Normal range
Total Protein (g/dL)	7.7	8.4	7.7	5.3	6.6–8.1
Albumin (g/dL)	4.6	4.9	4.6	3.1	4.1–5.1
T-Bil (mg/dL)	0.9	0.6	0.9	1.2	0.4–1.5
AST (IU/L)	33	25	39	170	13–30
ALT (IU/L)	65	56	95	254	10–42
LDH (IU/L)	168	122	162	500	124–222
γ-GTP (IU/L)	133	138	146	107	31–64
CPK (IU/L)	96	48	60	1312	59–248
BUN (mg/dL)	24.7	25.7	48.1	78.2	8–20
Creatinine (mg/dL)	0.50	0.52	0.66	1.57	0.55–1.07
Sodium (mmol/L)	140	139	144	146	138–145
Potassium (mmol/L)	4.6	4.1	4.2	5.8	3.5–4.5
Chloride (mmol/L)	102	95	97	97	101–108
Glucose (mg/dL)	107	110	122	103	73–109
C-reactive protein (mg/dL)	0.02	0.03	2.4	0.12	0–0.14
Procalcitonin (ng/mL)	0.01	0.02	0.09	1.26	<0.05
White Blood Cell (×10^3^/μL)	4.8	4.8	5.1	16.7	3.3–8.6
Neutrophil (×10^3^/μL)	1.7	2.0	2.2	11.4	1.5–7.0
Hemoglobin (g/dL)	14.1	15.5	16.8	14.4	13.7–16.8
Platelet (×10^4^/μL)	30.2	26.7	21.1	14.6	15.8–34.8
TSH (µIU/mL)			0.001	0.001	0.35–4.94
Free T3 (pg/mL)			28.3	27.6	1.88–3.18
Free T4 (ng/dl)			10.0	9.8	0.70–1.48
TRAb (IU/L)			30.6		–2.0
TsAb (%)			3020		–110
TgAb (IU/mL)			774.0		–28.0
TPOAb (IU/mL)			102.0		–16.0
BNP (pg/mL)			7.3	82.4	–18.4
PT-INR				1.68	0.85–1.15
Lactate (mmol/L)				3.6	0.5–1.9

**Figure 2:**
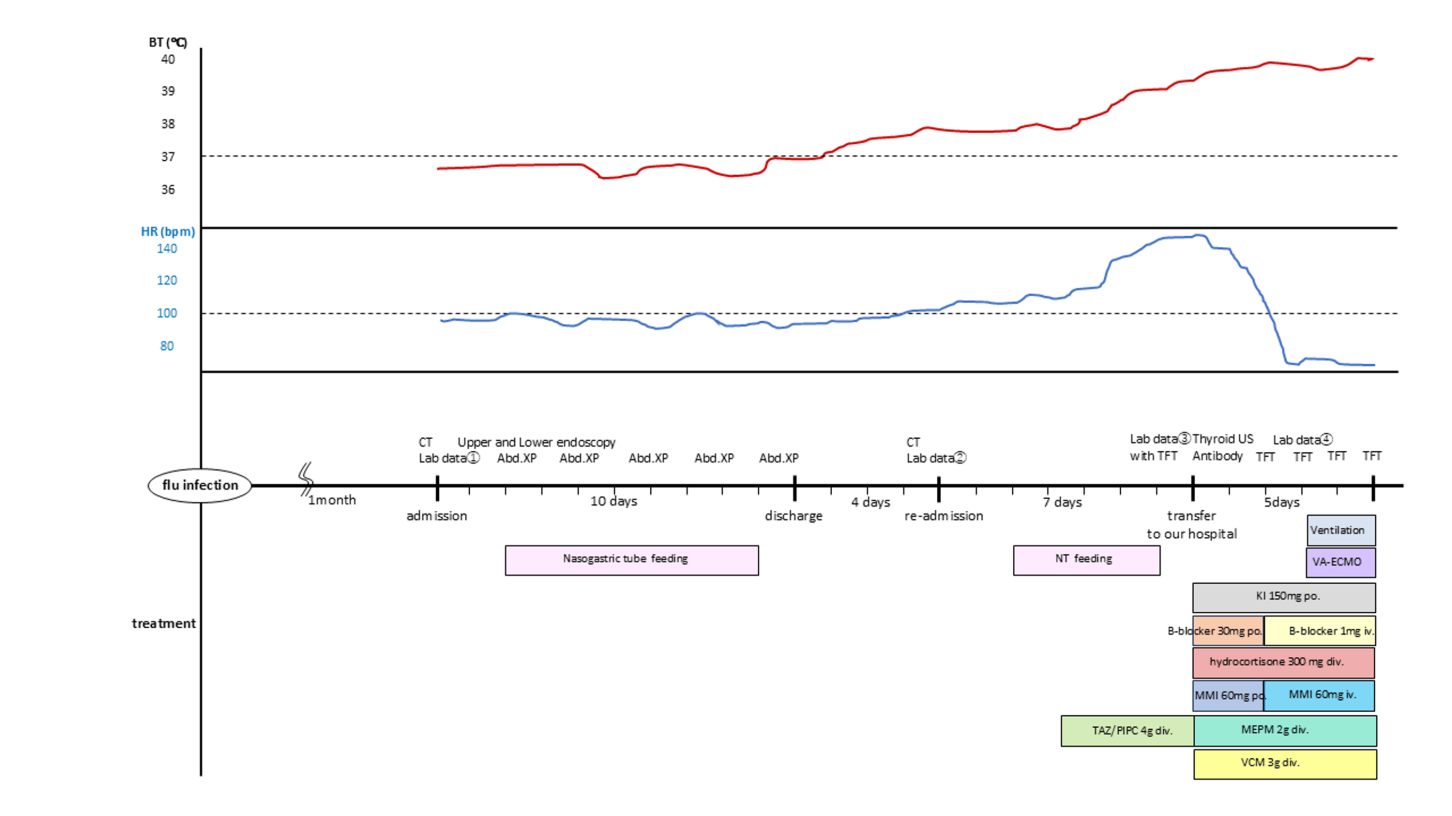
Clinical course BT, body temperature; HR, heart rate; bpm, beats per minute; CT, computed tomography; Abd. XP, abdominal X-ray; TFT, thyroid function test; US, ultrasound; NT, nasogastric tube; VA-ECMO, veno-arterial extracorporeal membrane oxygenation; KI, potassium iodide; po, per os; B-blocker, beta blocker; MMI, methimazole; TAZ/PIPC, tazobactam/piperacillin; MEPM, meropenem; VCM, vancomycin; Lab data①, day 30 of illness; Lab data②, day 44 of illness; Lab data③, day 51 of illness; Lab data④, day 54 of illness.

On transfer to our hospital, vital signs revealed tachycardia (142 beats/min) and hypotension (88/50 mm Hg). He had a goiter on physical examination. Thyroid ultrasonography demonstrated diffuse thyroid enlargement with increased vascularity. Although thyroid-associated ophthalmopathy was not observed, the presence of positive thyroid autoantibodies led to the diagnosis of Graves’ disease. He fulfilled both the Burch-Wartofsky Point Scale [9] and the diagnostic criteria of the Japan Thyroid Association [10] for thyroid storm. Following methimazole administration, treatment with potassium iodide, β-blocker, and corticosteroids was initiated. From day 2, oral therapy became impossible because of vomiting and diarrhea, and methimazole and β-blocker were administered intravenously. On day 3, he developed shock and signs of multiorgan failure (Figure 2). Tachycardia and atrial fibrillation were observed, which subsequently progressed to ventricular tachycardia, resulting in cardiac arrest. Intubation, mechanical ventilation, and extracorporeal membrane oxygenation were initiated. Despite continued intravenous methimazole and corticosteroid therapy, multiorgan failure progressed, and the patient died five days after admission. Thyroid function did not improve during hospitalization.

## Discussion

Thyroid storm is a rare condition, with an incidence of 0.57-1.0 per 100,000 persons in the general population [5,11]; however, it carries a high mortality rate of 10-30% [3,12,13], and the most common cause of death is multiorgan failure [7,14]. Accordingly, thyroid storm represents a life-threatening endocrine emergency.

Clinically, patients typically present with fever, altered mental status, palpitations, and weight loss [14,15], in which case the diagnosis is relatively straightforward. However, when only atypical manifestations are present, the condition is more likely to be overlooked at the initial presentation, resulting in diagnostic delay [1,13,16]. In older adults, the so-called apathetic storm, characterized by the absence of classic hyperadrenergic features, may occur [11,17] and contribute to delayed recognition and increased mortality [2]. Nevertheless, atypical presentations have also been seen in younger individuals.

This case illustrates several clinically important points. First, thyroid storm may present predominantly with gastrointestinal symptoms, including nausea, vomiting, and weight loss, without prominent fever or neuropsychiatric disturbance. Such atypical presentations may obscure early consideration of thyroid dysfunction. Gastrointestinal symptoms appeared following an episode of influenza infection, and it is possible that hyperthyroidism due to Graves’ disease developed around this time. Counting from this point, approximately 51 days elapsed before the diagnosis of Graves’ disease was established. Although the exact timing of progression from thyrotoxicosis to thyroid storm remains unclear, thyroid storm was diagnosed on the same day as the diagnosis of Graves’ disease, and treatment was initiated immediately.

Second, radiological findings suggestive of SMA syndrome contributed to diagnostic anchoring. While imaging findings were compatible with SMA syndrome, they did not fully explain the progressive systemic deterioration. Anchoring bias - premature fixation on an initial diagnosis - may delay reassessment when the clinical course evolves. On repeat CT at readmission, the findings suggestive of SMA syndrome had resolved. Therefore, the SMA syndrome-like findings observed on the initial CT were considered secondary changes associated with rapid weight loss.

Third, early thyroid function testing in patients with persistent unexplained gastrointestinal symptoms accompanied by tachycardia or systemic instability may facilitate timely diagnosis. When the diagnosis is established promptly, and guideline-directed therapy is initiated, mortality has been reported to decrease to 1.2-4.7% [4,5]. In contrast, delayed diagnosis or inappropriate management increases mortality to approximately 50% [4].

## Conclusions

In summary, thyroid storm may masquerade as a primary gastrointestinal disease, and radiological findings may be misleading. Lack of hyperthermia and neuropsychiatric symptoms does not rule out thyroid storm. Thyroid screening should be undertaken in patients presenting with diarrhea, weight loss, persistent vomiting, or unexplained liver function test abnormalities.

## References

[1] Farooqi S, Raj S, Koyfman A, Long B (2023). High risk and low prevalence diseases: thyroid storm. Am J Emerg Med.

[2] El-Menyar A, Khan NA, Elmenyar E (2025). Thyroid storm-induced cardiovascular complications and modalities of therapy: up-to-date review. World J Crit Care Med.

[3] Akamizu T (2018). Thyroid storm: a Japanese perspective. Thyroid.

[4] Furukawa Y, Tanaka K, Isozaki O (2024). Prospective multicenter registry-based study on thyroid storm: the guidelines for management from Japan are useful. J Clin Endocrinol Metab.

[5] Galindo RJ, Hurtado CR, Pasquel FJ, García Tome R, Peng L, Umpierrez GE (2019). National trends in incidence, mortality, and clinical outcomes of patients hospitalized for thyrotoxicosis with and without thyroid storm in the united states, 2004-2013. Thyroid.

[6] Idrose AM (2015). Acute and emergency care for thyrotoxicosis and thyroid storm. Acute Med Surg.

[7] Ono Y, Ono S, Yasunaga H, Matsui H, Fushimi K, Tanaka Y (2016). Factors associated with mortality of thyroid storm: analysis using a national inpatient database in Japan. Medicine (Baltimore).

[8] Merrett ND, Wilson RB, Cosman P, Biankin AV (2009). Superior mesenteric artery syndrome: diagnosis and treatment strategies. J Gastrointest Surg.

[9] Burch HB, Wartofsky L (1993). Life-threatening thyrotoxicosis: thyroid storm. Endocrinol Metab Clin North Am.

[10] Akamizu T, Satoh T, Isozaki O (2012). Diagnostic criteria, clinical features, and incidence of thyroid storm based on nationwide surveys. Thyroid.

[11] Thiyagarajan A, Platzbecker K, Ittermann T, Völzke H, Haug U (2022). Estimating incidence and case fatality of thyroid storm in Germany between 2007 and 2017: a claims data analysis. Thyroid.

[12] Chiha M, Samarasinghe S, Kabaker AS (2015). Thyroid storm: an updated review. J Intensive Care Med.

[13] Swee du S, Chng CL, Lim A (2015). Clinical characteristics and outcome of thyroid storm: a case series and review of neuropsychiatric derangements in thyrotoxicosis. Endocr Pract.

[14] Satoh T, Isozaki O, Suzuki A (2016). 2016 guidelines for the management of thyroid storm from the Japan Thyroid Association and Japan Endocrine Society (first edition). Endocr J.

[15] Pearce EN (2006). Diagnosis and management of thyrotoxicosis. BMJ.

[16] Radhi MA, Natesh B, Stimpson P, Hughes J, Vaz F, C Dwivedi R (2020). Thyroid storm in head and neck emergency patients. J Clin Med.

[17] Sabir AA, Sada K, Yusuf BO, Aliyu I (2016). Normothermic thyroid storm: an unusual presentation. Ther Adv Endocrinol Metab.

